# ^1^H NMR Metabolic Profile of Scyphomedusa *Rhizostoma pulmo* (Scyphozoa, Cnidaria) in Female Gonads and Somatic Tissues: Preliminary Results

**DOI:** 10.3390/molecules25040806

**Published:** 2020-02-13

**Authors:** Federica Angilè, Laura Del Coco, Chiara Roberta Girelli, Lorena Basso, Lucia Rizzo, Stefano Piraino, Loredana Stabili, Francesco Paolo Fanizzi

**Affiliations:** 1 Department of Biological and Environmental Sciences and Technologies (Di.S.Te.B.A.), University of Salento, via Monteroni, 73100 Lecce, Italy; federica.angile@unisalento.it (F.A.); laura.delcoco@unisalento.it (L.D.C.); chiara.girelli@unisalento.it (C.R.G.); lorena.basso@unisalento.it (L.B.); stefano.piraino@unisalento.it (S.P.); loredana.stabili@irsa.cnr.it (L.S.); 2 CoNISMa, Piazzale Flaminio, 9, 00196 Roma, Italy; lucia.rizzo@szn.it; 3 Integrative Marine Ecology, Stazione Zoologica Anton Dohrn, Villa Comunale, 80121 Napoli, Italy; 4 Water Research Institute of the National Research Council, (IRSA-CNR), Via Roma 3, 74123 Taranto, Italy; 5 CIRCMSB, Piazza Umberto I, 1, 70121 Bari, Italy

**Keywords:** *Rhizostoma pulmo*, jellyfish, female gonads, umbrella, oral arms, metabolomic, ^1^H NMR Spectroscopy

## Abstract

The Mediterranean basin is one of the regions heavily affected by jellyfish bloom phenomena, mainly due to the presence of scyphozoans, such as *Rhizostoma pulmo*. The jellyfish have few natural predators, and their bodies represent an organic-rich substrate that can support rapid bacterial growth with great impact on the structure of marine food webs. In Asiatic countries, jellyfish are widely studied for their health benefits, but their nutritional and nutraceutical values still remain poorly characterized. In this study, the differences in the ^1^H NMR spectroscopy metabolic profiles of *R. pulmo* female gonads and body fractions (including umbrella and oral arms), in different sampling periods, were studied. For each body compartment both lipid and aqueous extracts were characterized and their ^1^H NMR metabolic profiles subjected to multivariate analysis. From a statistical analysis of the extracts, a higher contents of ω-3 polyunsaturated fatty acids (PUFAs), amino acid and osmolytes (homarine, betaine, taurine) with important roles in marine invertebrates were observed in female gonads, whereas umbrella and oral arms showed similar metabolic profiles. These results support a sustainable exploitation of the jellyfish for the extraction of bioactive compounds useful in nutraceutical, nutricosmetics, and functional food fields.

## 1. Introduction

Jellyfish represent one of the most widespread components of marine ecosystems and their presence as a natural phenomenon is strictly related to seasonality. In recent years, numerous outbreaks (or blooms) of jellyfish have been recorded in many marine areas worldwide [1]. Jellyfish blooms have caused many negative consequences for the economy, fish population, and community, such as a decrease of coastal tourism, the reduction of fish abundance through competition or predation, increased mortalities of farmed fish, and obstruction of cooling intakes [2,3]. Among marine ecosystems, the Mediterranean Sea is one of the most heavily affected by jellyfish blooms, mainly due to the presence of scyphozoans, in particular of *Rhizostoma pulmo* (Macri, 1778), known as “white barrel” or “sea lung” [4]. This species has been the most abundant along the Mediterranean coasts in recent years, showing inter-annual fluctuations in population density [5,6]. During blooming years in the Mediterranean Sea, *R. pulmo* occurred in large numbers in several places, for example, the Northern and Southern Adriatic Sea, the Ionian Sea, in the Eastern and Western Mediterranean, as well as in the Black Sea [5]. While the impact jellyfish blooms have on marine ecosystems is generally considered negatively, jellyfish species are very important, on the other hand, as foodstuff and for their therapeutic properties, especially in Asiatic countries [3]. In the first decade of the 21st century, over 2000 active compounds have been isolated from cnidarians and mainly used as drugs in therapy of human diseases [7,8]. In jellyfish, these properties seem to be attributable essentially to collagen, the main protein component of jellyfish connective tissue [3,9]. Furthermore, genomic, molecular, and ultra-structural studies have shown that the collagen of these invertebrates, in particular *R. pulmo*, is similar to that of mammalian type I collagen and could replace bovine or human collagens in the biomedical field [10]. In addition, other studies have successfully demonstrated that the collagen of gelatinous zooplankton could represent a new source of advantageous products for human consumption, such as collagen peptides and hydrolysate, used in medicine and food industries [3,9,11,12,13,14,15,16,17]. Finally, other studies have shown that jellyfish used as feed have a great value without toxic effects on chickens and pigs, as well as some commercial fishes [14,18]. Despite jellyfish representing a multimillion dollar seafood business in Asiatic countries and their pro-health value for metabolites composition and properties in low calorie diet [3,19,20], edible jellyfish metabolomic profiles are poorly characterized. Recently [4], the metabolic profiles of specific body fractions (ovaries) of *R. pulmo* have been investigated for the first time by ^1^H NMR spectroscopy, together with their biochemical composition and antimicrobial properties. Previous results showed that *R. pulmo* ovaries and oocytes could represent a promising source of bioactive compounds for different applications mainly in the pharmaceutical field or as specialty feed [4]. With the aim of defining a sustainable exploitation pilot system of distinct jellyfish body fractions for different purposes (e.g., food, feed), this work focuses on the metabolic profile analysis for three different body compartments of *R. pulmo* in three different periods of the year. In particular, the differences in amino acids, other organic molecules (with important biochemical roles), and lipid (especially in ω-3 PUFAs content) composition were evaluated in female gonads, and umbrella and oral arms in different sampling periods (March, May, July) by a ^1^H NMR-based metabolomic approach.

## 2. Results

### 2.1. NMR Spectroscopy

**Figure 1:**
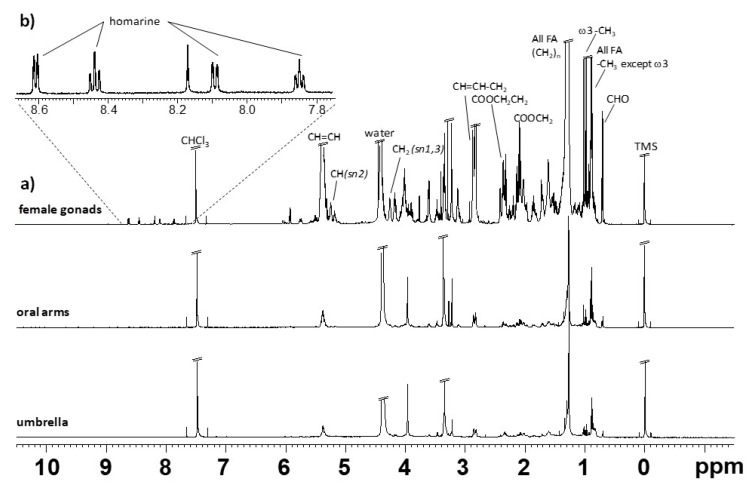
Typical ^1^H NMR spectrum obtained at 600 MHz of CD_3_OD/CDCl_3_ lipid extract of *R. pulmo*. (**a**) Full spectrum of three body compartments, and (**b**) expansion spectrum at high frequency regions obtained only for the female gonads extracts.

**Figure 2:**
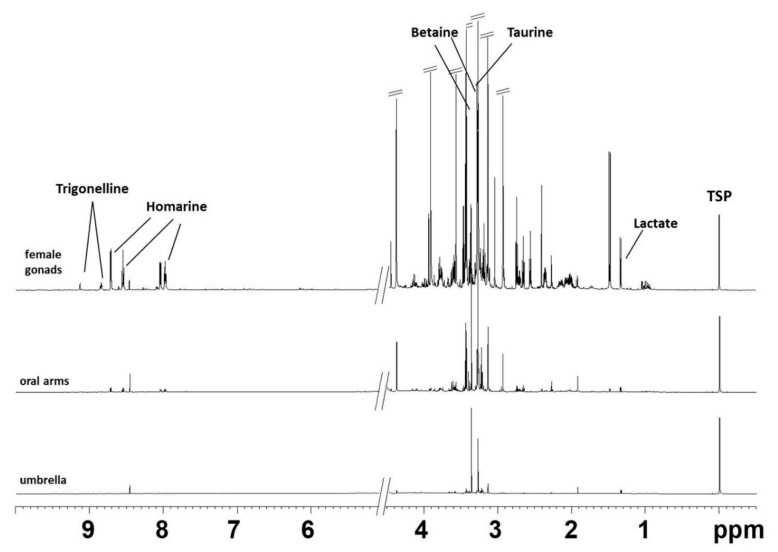
Typical ^1^H NMR spectrum obtained at 600 MHz of D_2_O aqueous extract of different body compartments of *R. pulmo* (female gonads, oral arms, umbrella).

Representative 1D ^1^H NMR spectra of lipid and aqueous extracts of female gonads and somatic tissues (umbrella and oral arms) of *R. pulmo* are shown in Figure 1 and Figure 2, respectively. All the ^1^H NMR spectra of lipid extracts of body fractions showed the characteristic signals of saturated (SFAs), mono- (MUFAs), di- (DUFAs) and polyunsaturated (PUFAs) fatty acids, triglycerides (TG), and minor components, such as sterols (cholesterol, CHO), phosphatidycholine (PC) and phosphatidylethanolamine (PE) (Table 1, Figure 1), according to the literature data [21,22,23,24] and previously reported by Stabili et al. [4]. Interestingly, homarine was clearly identified only in the female gonad lipid extracts, together with a more intense concentration of the fatty acid content with respect to somatic tissues, as already reported in the literature for other scyphozoan jellyfish species [25,26]. Analogously, for the ^1^H NMR aqueous spectra, the metabolites were assigned on the basis of analysis of 2D NMR spectra (2D ^1^H J-RES, ^1^H COSY, ^1^H ^13^C HSQC, and HMBC) and by comparison with published data [21,22,23,24,27,28]. The NMR aqueous spectra of *R. pulmo* body compartments were characterized by essential amino acids, such as branched-chain isoleucine, leucine and valine, threonine, and no-essential amino acids (alanine, proline, glutamate, β-alanine, glycine, tyrosine). Moreover, organic acids (such as lactate, acetate, formate, succinate) and other molecules with important biochemical functions, for instance, betaine, taurine (2-aminosulphonic acid), and homarine (N-methylpicolinic acid), already known in marine invertebrates [28] and essentially acting as osmolytes [29] were also identified (Table 2, Figure 2). Homarine signals were observed in both lipid and aqueous extracts. Furthermore, few metabolites were observed in the aqueous body extract portions. These are essentially carboxylic acid derivatives, including lactate, acetate, formate, and osmolytes, such as betaine, taurine, hypotaurine, and homarine.

### 2.2. Statistical Analysis

Multivariate statistical analysis (MVA) was applied to the whole NMR datasets (bucket tables obtained both for lipid and aqueous extracts), focusing on the possible differences in metabolite content of different body compartments of *R. pulmo* (paying specific attention on female gonads) collected in different sampling periods. The unsupervised analyses (principal component analysis, PCA), performed on the whole data for both the lipid and aqueous extracts, revealed a clear differentiation between the female gonads and the somatic tissues (Figure S1 in the SI). In particular, the potential effect of the sampling periods gave an interesting hint for the sample distribution of female gonads in the PCA score scatterplot. In order to deeply analyze this aspect, a further investigation was performed considering separately the female gonads, umbrella, and oral arms, using both unsupervised (PCA) and supervised multivariate analytical (orthogonal partial least squares discriminant analyses, OPLS-DA) methods. The robustness of the statistical models was tested by cross-validation default method (seven-fold) and further evaluated with a permutation test (400 permutations). The quality of the statistical models (in particular the total variations in the data and the internal cross-validation) was described by R^2^ (cross validation) and Q^2^ (predictive ability) parameters, as described in Section 4.4 (Statistical Analysis).

#### 2.2.1. The Potential Effect of Sampling Period on *R. pulmo* Metabolomic Profile

##### Lipid Extracts

A MVA was performed with the aim to differentiate, according to the sampling periods, the lipid extracts of female gonads, and other body portions.

**Figure 3:**
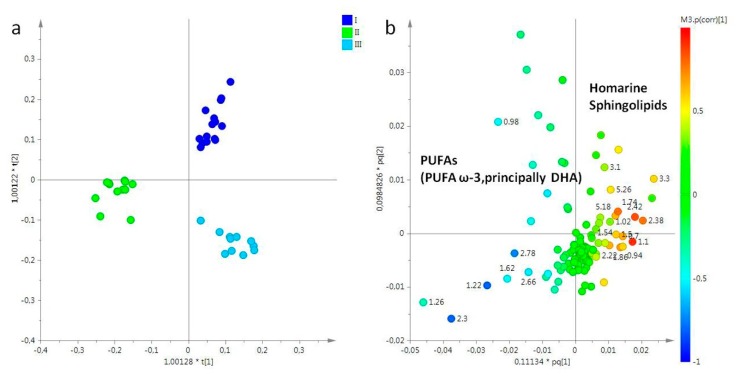
(**a**) OPLS-DA t[1]/t[2] scores plot of for lipid extracts of female gonads of *R. pulmo* collected in different sampling periods: blue circle, March sampling (I); green circle, May sampling (II); sky blue circle, July sampling (III). (**b**) Loading plot for the model; the variables indicated ppm in the ^1^H NMR spectrum.

Lipid extracts obtained from female gonads were studied considering three sampling periods (March, May, and July). In particular, the PCA model was obtained considering five principal components, with more than 90% of the explained variance, and R^2^X = 0.96 and Q^2^ = 0.91. In the PCA score plot, samples collected in March (I) and July (III) were clearly separated from samples collected in May (II) (data not shown). In order to improve the separation among the different sampling periods, and clearly define the metabolites responsible for the differentiation, the OPLS-DA was also performed. In the OPLS-DA score plot (2+3+0, R^2^X = 0.95, R^2^Y = 0.93, Q^2^ = 0.874), all three groups were clearly distinct. In particular, samples of March (I) and July (III) resulted in positive values of the predictive t[1] component (between 0 and 0.20), while samples collected in May (II) were found a negative values (between −0.1 and −0.3) of the same t[1] predictive component. Moreover, samples from the first sampling period was clearly differentiated from samples obtained in the third sampling (July), along the second component t[2] (Figure 3a). From analysis of loading plot for the model (Figure 3b), samples collected in March (I) and July (III) were characterized by a higher content of homarine and sphingolipids respectively, whereas high levels of PUFAs, including PUFA ω-3 (principally docosahexaenoic acid, DHA), were characteristic of samples collected in May (II).

**Figure 4:**
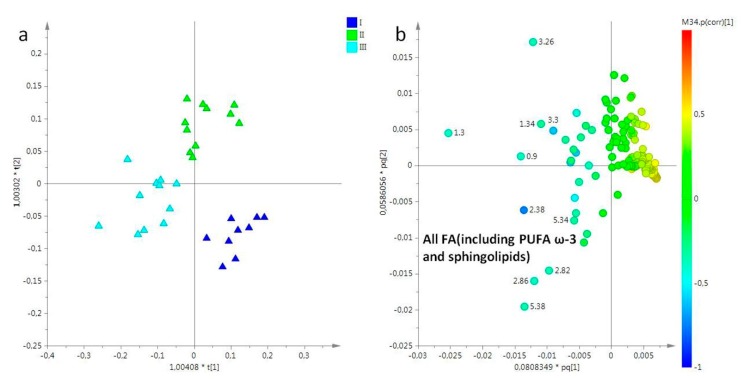
(**a**) OPLS-DA t[1]/t[2] scores plot of for lipid extracts of umbrella of *R. pulmo* collected in different sampling periods: blue triangle, March sampling (I); green triangle, May sampling (II); sky blue triangle, July sampling (III). (**b**) Loading plot for the model; the variables indicated ppm in the ^1^H NMR spectrum.

Moreover, MVA was then applied in lipid extracts obtained from the umbrellas of *R. pulmo* jellyfish. Additionally, in this case, three sampling periods were considered. A PCA model was obtained using three components (77%, 10%, 5% for t[1], t[2], t[3], respectively), with 92% of the total variance explained. The t[1]/t[2] score plot showed a good separation between three considered sampling periods (data not shown). This trend was confirmed by the corresponding supervised analysis. In particular, in the OPLS-DA score plot (2+3+0 R^2^X = 0.95, R^2^Y = 0.804, Q^2^ = 0.634), samples were well separated along the predictive component t[1], depending from the sampling periods. The samples collected in March and May were found at positive values of predictive t[1] component, while samples collected in July were at negative values of the same component (Figure 4a). Examining the corresponding loading plot for the model emerged that the samples collected in July were characterized by high levels of FA (considering all FA, including PUFA ω-3 and sphingolipids) with respect to other sampling periods (Figure 4b).

**Figure 5:**
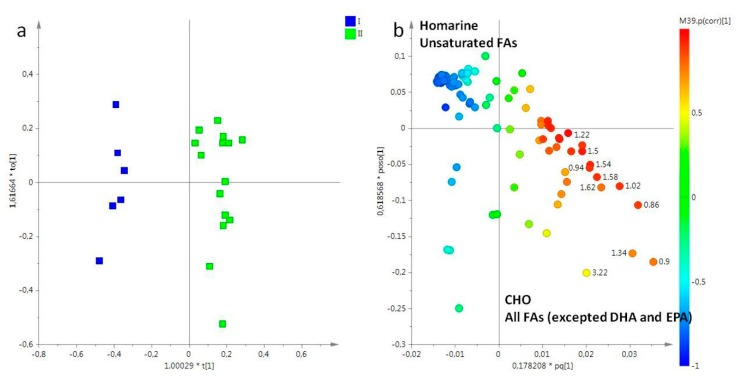
(**a**) OPLS-DA t[1]/t[2] scores plot of for lipid extracts of tentacles of *R. pulmo* collected in different sampling periods: blue box, March sampling (I); green box, May sampling (II). (**b**) Loading plot for the model; the variables indicated ppm in the ^1^H NMR spectrum.

Only two sampling periods (March and May) for both lipid and aqueous oral arm extracts were considered. In the PCA model obtained from oral arm lipid fractions, the t[1]/t[2] PCA score plot showed a good separation of samples (data not shown), with four principal components enough to describe the 90% of total variance of the entire dataset, and R^2^X = 0.98 and Q^2^ = 0.97. As already observed in the preliminary PCA, also in this OPLS-DA score plot (1+3+0, R^2^X = 0.98, R^2^Y = 0.94, Q^2^ = 0.90), samples appeared clearly differentiated along the first t[1] predictive component (Figure 5a). In details, the model showed that samples obtained from oral arms collected in March (I) were found at negative values of the first predictive component t[1] (between −0.4 and −0.6), while samples collected in May (II) were positioned at positive values of same component (between 0 and 0.4). By the analysis of the loading plot for this model, it was possible define the original variables responsible of the observed separation (Figure 5b). Specifically, samples collected in the first sampling period (I, March) were characterized by a high relative content of homarine, and unsaturated fatty acids, whereas high levels of CHO, and all FAs, except DHA and eicosapentaenoic acid (EPA), were found in samples collected in May (II).

Interestingly, the existing differences between March and May sampling periods were highly marked for oral arms, especially when compared to differences related to the other two body compartments of the same sampling periods. These observations could suggest that the oral arms compartment was subjected to striking metabolic fluctuations, which could be probably due to physiological conditions of this jellyfish compartment. Obviously these preliminary results require a deeper level of investigation for a better knowledge of the observed pattern of *R. pulmo* oral arms which is beyond the aim of this work.

**Figure 6:**
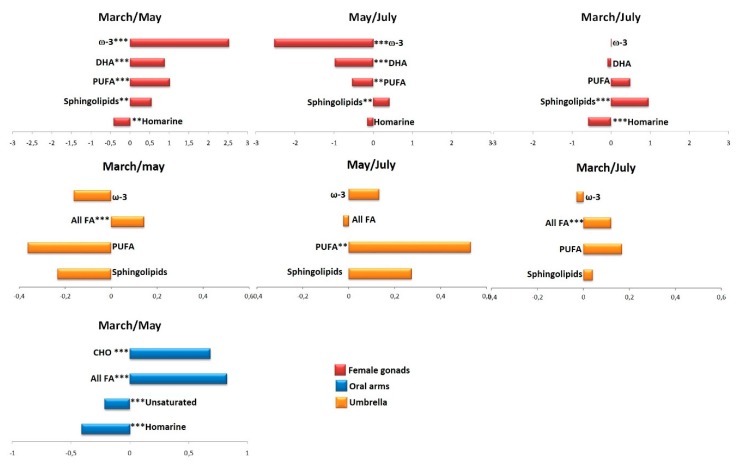
Variation in discriminating metabolites content for each compartments, among the different sampling (March, May, and July). Signif. codes: ‘***’ 0.001 ‘**’ 0.01 ‘*’ 0.05 ‘.’ 0.1 ‘ns’ 1.

Moreover, the variation in discriminating metabolites content for each body compartments, among the different sampling was calculated by the integration of selected distinctive unbiased NMR signals. In particular, signals corresponding to homarine (δ ^1^H 8.1), unsaturated fatty acids (δ ^1^H 5.3), all FA acids (δ ^1^H 1.26 and δ ^1^H 2.18), CHO (δ ^1^H 1.01), PUFA (δ ^1^H 2.86), sphingolipids (δ ^1^H 3.22), PUFA ω-3 (δ ^1^H 0.98), and DHA (δ ^1^H 2.38) were selected and integrated (Figure 6).

##### Aqueous Extracts

Despite the large biomasses, the dry weight of most Rhizostomeae jellyfish (Cnidaria, Scyphozoa) ranges from 2–5%, and is mainly composed of proteins, having carbohydrates and lipids as minor components [11]. For this reason, we also analyzed *R. pulmo* aqueous extracts. Multivariate statistical analysis was also performed on aqueous extracts of each body compartment of *R. pulmo* (female gonads, umbrella, oral arms).

**Figure 7:**
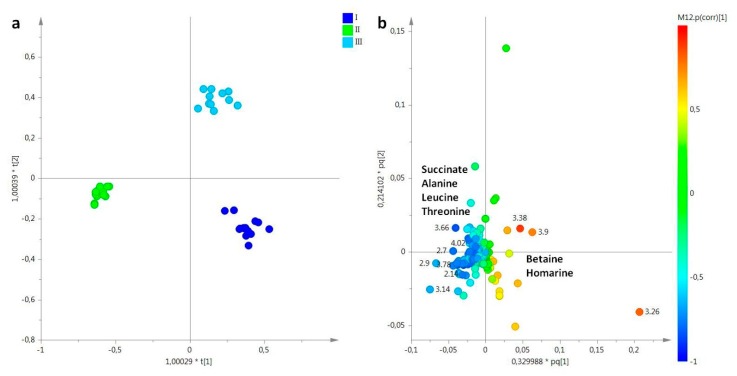
(**a**) OPLS-DA t[1]/t[2] scores plot of for aqueous extracts of female gonads of *R. pulmo* collected in different sampling periods: blue circle, March sampling (I); green circle, May sampling (II); sky blue circle, July sampling (III). (**b**) Loading plot for the model; the variables indicated ppm in the ^1^H NMR spectrum.

A first attempt, unsupervised analysis was performed in aqueous extracts of female gonads, obtaining a good model with R^2^X = 0.82 and Q^2^ = 0.70 and only three principal components described the 80% of total variance (t[1], t[2] and t[3] principal components explained 52.6%, 21%, and 8%, respectively). A certain degree of separation based on the sampling periods was observed in the t[1]/t[2] PCA scores plot, along the first principal component t[1], with samples from the second sampling (May, II) isolated from the other two sampling periods of March and July (data not shown). In order to refine the separation among the three groups, a supervised analysis was also performed. In the OPLS-DA score plot (2+4+0, R^2^X = 0.92, R^2^Y = 0.98, Q^2^ = 0.95, Figure 7a), samples collected in March (I) and in July (III) were again clearly separated from samples collected in May (II), especially along the first predictive component t[1]. By examining the loadings plot, a marked content of osmolytes was detected, as homarine was observed in the March (I) sampling and betaine was found in the March (I) and July (III) samplings, while a high relative content of succinate and amino acids, such as alanine, leucinem and threonine, characterized female gonads from the May (II) sampling (Figure 7b).

**Figure 8:**
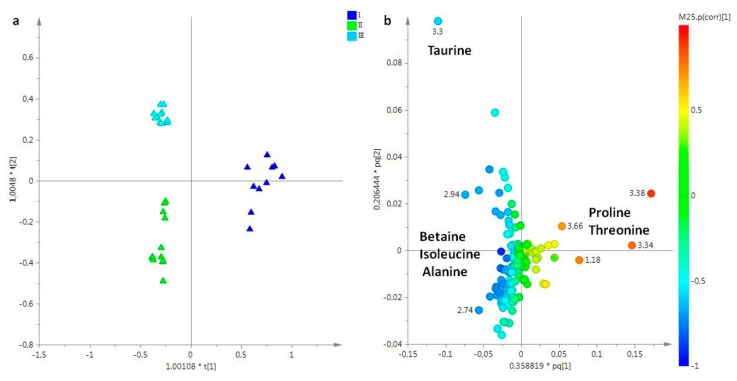
(**a**) OPLS-DA t[1]/t[2] scores plot of for aqueous extracts of umbrella of *R. pulmo* collected in different sampling periods: blue triangle, March sampling (I); green triangle, May sampling (II); sky blue triangle, July sampling (III). (**b**) Loading plot for the model; the variables indicated ppm in the ^1^H NMR spectrum.

Successively, the aqueous extracts of umbrella were deeply analysed. The t[1]/t[2] PCA score plot (R^2^X = 0.78, Q^2^ = 0.484) performed for umbrella aqueous extracts showed a good separation of samples along the t[1] principal component, with samples collected in May and July separated by samples collected in March (data not shown). In order to improve the observed separation and clearly define the metabolites responsible for the differentiation, a supervised OPLS-DA analysis was then applied providing a good model (2+2+0, R^2^X = 0.723, R^2^Y = 0.927, Q^2^ = 0.87) (Figure 8a). Umbrella aqueous samples collected in May (II) and in July (III) appeared at negative values of the predictive component t[1], whereas samples collected in March (I) were observed at positive values of the same component t[1] and quite scattered along orthogonal component t[1]. From the loading for this model (Figure 8b), molecular components responsible for the separation between the two groups could be observed. In the first sampling (I, March), a high relative content of amino acids, in particular proline and threonine was observed, while samples collected in the second sampling period (II, May) were characterized by relative higher levels of betaine and amino acid, such as isoleucine and alanine. The third sampling period was characterized by high relative content of taurine, when compared with the other two periods.

**Figure 9:**
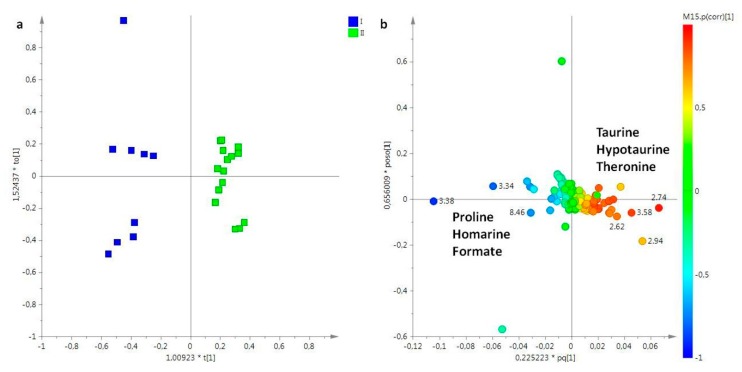
(**a**) OPLS-DA t[1]/t[2] scores plot of for aqueous extracts of oral arms of *R. pulmo* collected in different sampling periods: blue box, March sampling (I); green box May sampling (II). (**b**) Loading plot for the model; the variables indicated ppm in the ^1^H NMR spectrum.

Similarly to oral arms lipid extracts, for aqueous extracts only March and May sampling periods were considered. The unsupervised analysis gave a good model R^2^X = 0.89 and Q^2^ = 0.42 and only three components described more than 74% of the total variance (36%, 30%, and 8.8% for t[1], t[2], and t[3], respectively). In the corresponding t[1]/t[2] PCA score plot, the samples collected in March and May (I and II) were well separated, along the t[2] principal component (data not shown). The equivalent OPLS-DA model (1+1+0, R^2^X = 0.54, R^2^Y = 0.95, Q^2^ = 0.91) improved the separation among samples along the predictive component t[1], from negative (between −0.2 and −0.6) to positive values (between 0.2 and 0.4) for samples collected in March (I) and May (II), respectively (Figure 9a). From the analysis of the loading-plot for the model (Figure 9b), a high relative content of proline, homarine, and formate was found in oral arms of March period, whereas a higher relative content of taurine, hypotaurine, threonine, and glycine was observed in samples collected in May.

**Figure 10:**
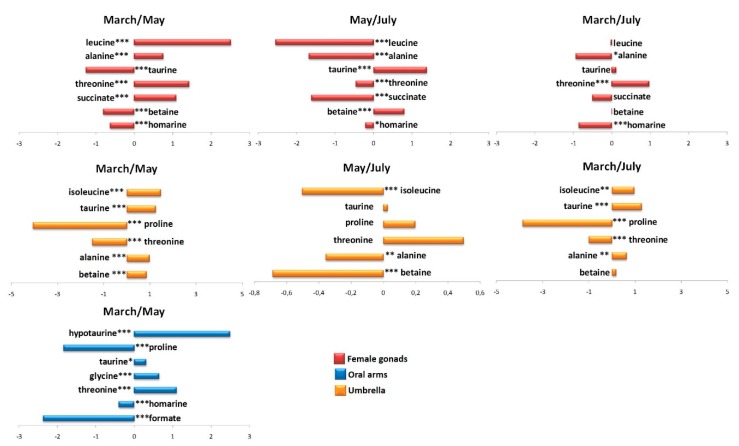
Variation in discriminating metabolites content for each compartments, among the different sampling (March, May, and July). Signif. codes: ‘***’ 0.001 ‘**’ 0.01 ‘*’ 0.05 ‘.’ 0.1 ‘ns ‘ 1.

As in lipid extracts, for aqueous extracts the variation in discriminating metabolite content for each compartment among the different sampling was calculated by the integration of selected distinctive unbiased NMR signals. In particular, signals corresponding to formate (δ ^1^H 8.46), homarine (δ ^1^H 4.37 and 7.98), threonine (δ ^1^H 3.66), glycine (δ ^1^H 3.57), taurine (δ ^1^H 3.27, and 3.41), proline (δ ^1^H 3.34), hypotaurine (δ ^1^H 2.62), isoleucine (δ ^1^H 1.98), alanine (δ ^1^H 1.50), betaine (δ ^1^H 3.90), succinate (δ ^1^H 2.41), and leucine (δ ^1^H 0.98) were selected and integrated (Figure 10).

## 3. Discussion

Recently, scientific interest has focused on bioactive compound research from marine organisms, which represent a possible source of healthy food and molecules with potential antibacterial activity [3,4]. In a previous work, the prospect to exploit *R. pulmo* ovaries as source of bioactive compounds for different applications in pharmaceutical and nutrition fields was discussed [4]. On the basis of these results, the metabolic profiles of *R. pulmo* body fractions (female gonads, umbrella, and oral arms) were analysed in different sampling periods by ^1^H NMR spectroscopy. The present study has the aim of laying the groundwork for sustainable exploitation of *R. pulmo* body compartments for different purposes useful for human activity and health.

In both extracts, lipid and aqueous, a higher content of metabolites was identified in female gonads with respect to other two compartments. The lipid extract analysis showed a high content of ω-3 PUFAs, as also observed by other authors in different species of scyphozoan jellyfish, such as *Aurelia* sp. [3,4,8,30]. The high content of PUFAs (essentially ω-3 and ω-6 PUFA) in these organisms could represent an important source as essential dietary nutrients for marine invertebrates and fish, since these cannot synthesize PUFA de novo from saturated and monounsaturated fatty acids [31]. Moreover, as reported in the literature, ω-3 PUFAs, especially DHA and EPA, are abundant in the gonads of marine invertebrates [32], including also scyphomedusae, as these molecules are required in the gonad maturation [33], larval growth of fish [34], reproduction processes, and for egg quality [4,35], regulation of cellular movement, gonadal metabolism of lipids, and fusion capacity [36]. Indeed, the lipid content strongly influenced the composition of fish eggs [37]. Moreover, it has also been reported that, in different species, such as *Octopus vulgaris*, the lipid gonad metabolism was regulated by the PUFA content [36]. In other organisms, such as crustaceans and molluscs, PUFAs are also involved in prostaglandin and hormone production and ionic flux regulation [4]. In some jellyfish species, such as *Aurelia* sp. and *R. pulmo* ovaries, EPA and DHA fatty acids (FAs) were detected, where palmitic and stearic fatty acids (16:0 and 18:0, respectively), EPA, AA, and DHA (eicosapentaenoic, arachidonic, and docosahexaenoic acids, respectively) constituted around 66% of the total FAs [38,39]. Since antioxidant and anti-inflammatory properties of ω-3 PUFAs, DHA, and EPA are suitable for treatments of neuro-inflammation-induced memory deficits and mental health [40,41], the exploitation of ω-3 PUFAs found in DHA and EPA of the *R. pulmo* gonads as sources of these compounds could be promising in pharmaceutical and nutraceutical fields. The observed differences in lipid content might be due to a variation in the size and age of individuals and/or the temperature of the living biota and habitat, as already observed in *Aurelia aurita* [38]. In particular, Lucas et al. [42] summarized the most common life history patterns in temperate waters explaining that juvenile medusa (ephyrae) are liberated in late winter and develop into medusae in the spring. As the temperature increases, the medusae reach the maximum size of 20–30 cm bell diameter [42]. During summer, the medusae start to shrink as they release eggs. Their life span is mostly 4–8 months. Accordingly, the life cycle of *R. pulmo* begins with a fertilized egg that develops into a planula and, hence, into a sessile and asexually-reproducing scyphistoma, which strobilates and releases swimming ephyrae that develop into medusae. Previous studies on the seasonal distribution of *R. pulmo* along the Catalan coast and the coastal lagoon on Mar Menor (NW Mediterranean) [43] evidenced that the adult medusae normally disappeared during January and March and that the juvenile *R. pulmo* (about 5–10 cm umbrella diameter) was observed at the beginning of May. On the basis of these observations, it is plausible that the observed differences in lipid concentration could be related to the maturity of the eggs in the collected jellyfish. In particular, in March the juveniles have not yet reached the sexual maturity and eggs have a different composition compared to those of April/May, when jellyfish are ready to mate. In this context, it is also intriguing to consider that in jellyfish, an increase in the amount of sunlight at dawn causes males and females to release sperm and eggs, respectively, into the water at the same time, therefore improving the chances of fertilization. In female jellyfish, the rise in the amount of sunlight falling on the cells surrounding the oocyte—the future egg—also stimulates the final steps in the process of egg production [44,45,46]. It should be noted that at our latitude (Ionian Sea, 40°25.7′ N, 16°53.1′ E; Italy) this situation could be reached just starting from May. After the spawning period the female gonads have low contents of unfertilized eggs and this could be reflected in the observed ^1^H NMR profiles. Interestingly, the results obtained in the present study are also in accordance with several studies on the biochemical composition of eggs in marine invertebrates, showing that mature eggs often are characterized by a high lipid and protein content.

In aqueous extracts, essential (leucine, isoleucine, threonine, valine) and non-essential amino acids (alanine, glycine, proline, and glutamate) were found as the most important, and dominant also in the gonads of other edible jellyfish, such as *Rhopilema esculentum* [30]. In particular, as already reported in the literature, amino acids are responsible for food flavor. Glycine, alanine, and proline are characteristics of a sweetish flavor [30], while glutamate was known to be responsible for a palatable flavor [30]. Other amino acids of particular interest in medicine, such as leucine, glycine, and tyrosine were found in female gonads of *R. pulmo* [30]. The aqueous extracts of female gonads were characterized by the presence of osmolytes, which could be involved in supporting the larval state until the metamorphosis, as found in Hydrozoa [4]. The higher presence of osmolytes in the gonads in March and July compared to May, when the ovaries are richer in amino acids, could also be related to the different stage of the egg maturation as already explained in the case of lipids.

## 4. Materials and Methods

### 4.1. Chemicals

All chemical reagents for analysis were of analytical grade: D_2_O, CDCl_3_, CD_3_OD (99.8% atom%D), TSP, 3-(trimethysilyl)-propionic-2,2,3,3,d4 acid, and tetramethylsilane, TMS (0.03 *v*/*v*%) and were purchased from Armar Chemicals (Döttingen, Switzerland).

### 4.2. Sampling

*R. pulmo* jellyfish were sampled in Ginosa Marina (Ionian Sea, 40°25.7′ N, 16°53.1′ E; Italy) as already reported in Stabili et al. [4]. Samplings were performed in three periods: March (I), May (II), and July (III) in 2017, respectively. Briefly, the specimens of *R. pulmo* adult medusae were sampled and immediately washed into the laboratory with filtered-sterilized seawater (0.2 μm, Millipore), to remove the mucus layer produced during transport. The different body fractions (female gonads, umbrella) were separated, frozen a −80 °C and lyophilized. Ovaries appeared from pink to orange, with easily distinguishable eggs. When gender determination was uncertain visually, a small piece of gonad tissue was removed and examined under a stereomicroscope. The ovaries were carefully dissected with microscissors at the stereomicroscope to avoid loss of gonadic tissue or accidental inclusion of subumbrellar or exumbrellar tissues. Each gonad was then divided in aliquots. Aliquots were lyophilized and frozen at −80 °C in liquid nitrogen until NMR analysis. In particular, for each sampling period, four specimens were considered for a total of 12 samples, and for every analyzed body compartment three technical replicates were assessed.

On March and May the oral arms of the jellyfish were also analyzed. This compartment was not taken into consideration in July considering that in this period jellyfish are involved in reproduction and this compartment is rich in eggs. In some jellyfish species, indeed eggs are attached to “brood pouches” on the upper part of the female’s arms, surrounding the mouth. In other species, the female harbors the eggs inside her mouth, and the male’s sperm swim into her stomach; the fertilized eggs later leave the stomach and attach themselves to the female’s arms.

The lipid and aqueous extracts samples of *R. pulmo* were prepared according to a modified Bligh and Dyer extraction method [4,47,48]. For each body compartment sample, lyophilized material (~100 mg) was added of 400 μL chloroform, 400 μL methanol, and 400 μL deionized filtered water. The solution was mixed and placed on an ice bath for 10 min before centrifugation at 10,000 rpm for 20 min at 4 °C. The two phases obtained, polar and lipophilic, were separated and dried by a SpeedVac concentrator (SC 100, Savant, Ramsey, MN, USA). The lipid extracts were dissolved in 700 μL of CD_3_OD/CDCl_3_ (1:2 mix) containing 0.03% *v*/*v* tetramethylsilane (TMS, δ = 0.00) as the internal standard. The polar extracts were dissolved in 160 μL 0.2 M Na_2_HPO_4_/NaH_2_PO_4_ buffer (pH 7.4) and 540 μL D_2_O containing 3-(trimethylsilyl)-propionic-2,2,3,3-d4 acid (TSP δ = 0.00) as the internal standard. Lipid and aqueous extracts were then transferred into 5 mm NMR tubes for NMR analysis.

### 4.3. NMR Measurements

All measurements were performed on a Bruker Avance III 600 Ascend NMR spectrometer (Bruker, Ettlingen, Germany), operating at 600.13 MHz for ^1^H observation, equipped with a z axis gradient coil and automatic tuning-matching (ATM). Experiments were acquired at 300 K in automation mode after loading individual samples on a Bruker Automatic Sample Changer, interfaced with the software IconNMR (Bruker). For each aqueous extracts a 1D sequence with pre-saturation and composite pulse for selection (zgcppr Bruker standard pulse sequence) was acquired, with 64 transients, 16 dummy scans, 5 s relaxation delay, size of FID (Free Induction Decay) of 64,000 data points, a spectral width of 12,019.230 Hz (20.0276 ppm) and an acquisition time of 2.73 s, using TSP, 3-(trimethysilyl)-propionic-2,2,3,3,d4 acid (δ = 0.00) as the internal standard. The lipid extracts were dissolved in 600 μL of CD_3_OD/CDCl_3_ (1:2 mix) and transferred to a 5-mm NMR tube, using tetramethylsilane (TMS, δ = 0.00) as the internal standard. The following parameters were used for ^1^H NMR spectrum: 64,000 data points, spectral width of 20.0276 Hz, 64 scans with a 5 s repetition delay, 90° power pulse (p1) 7.3 µs, power level 8.05 dB. The resulting FIDs were multiplied by an exponential weighting function corresponding to a line broadening of 0.3 Hz before Fourier transformation, automated phasing, and baseline correction. The metabolites were assigned on the basis of 2D NMR spectra analysis (2D ^1^H Jres, ^1^H COSY, ^1^H-^13^C HSQC, and HMBC) and by comparison with published data [21,22,23,27,28].

### 4.4. Statistical Analysis

The ^1^H NMR spectra were processed using Topspin 3.5 and Amix 3.9.13 (Analysis of Mixture, Bruker, Biospin, Italy), both for simultaneous visual inspection and the successive bucketing process. The NMR spectra performed for both lipid and aqueous extracts were segmented in rectangular fixed (0.04 ppm width) buckets and integrated by Amix software. The bucketing procedure was performed in the spectral range between 10.00 and 0.5 ppm, excluding the residual signals of non-deuterated solvents. In particular, the regions 7.75–7.05 ppm for chloroform and its carbon satellites, 4.75–3.65 ppm for water, and 3.41–3.33 ppm methanol were excluded in the case of NMR spectra of lipid extracts, while the region 5.00–4.60 ppm, corresponding to the residual water signal was excluded in the case of NMR spectra of aqueous extracts. The total sum normalization was applied to reduce small differences due to sample concentration and/or experimental conditions among samples. The two bucket tables (for lipid and aqueous extracts), obtained by alignment and successive buckets row reduced spectra, were submitted to multivariate data analysis (MVA). Each bucket row represents the entire NMR spectrum, with all the molecules in the sample. Moreover, each bucket in a buckets row reduced spectrum is labelled with the value of the central chemical shift for its specific 0.04 ppm width. The variables used as descriptors for each sample in chemometric analyses are the buckets. The description of statistical analyses refers to Pareto-scaled data obtained by dividing the mean-centered bucket values by the square root of the standard deviation [49]. Multivariate statistical analysis (MVA) was performed by using Simca-P version 14 (Sartorius Stedim Biotech, Umeå, Sweden). In particular, PCA (principal component analysis), PLS-DA, and OPLS-DA (Partial least squares and orthogonal partial least squares discriminant analyses, respectively) were applied to the data [49,50,51] to examine the intrinsic data variation. PCA is at the basis of the multivariate analysis [50] and usually performed to extract and display the systematic variation in a data matrix X formed by rows (the considered observations), in our case the samples and columns (the variables describing each sample) of the buckets from each NMR spectrum. A PCA model provides a summary, or overview, of all observations or samples in the data table. In addition, groupings, trends, and outliers can also be found. To further maximize the separation between sample classes (already observed by PCA), partial least-squares discriminant analysis (PLS-DA) was then applied. The PLS-DA is the regression extension of PCA, which gives the maximum covariance between the measured data (X variable, matrix of buckets related to metabolites in NMR spectra) and the response variable (Y variable, matrix of data related to the class membership). In this work, the PLS-DA method was also performed in order to justify the number of components used in the OPLS-DA model [50,51,52,53]. The OPLS-DA analysis is a modification of the usual PLS-DA method which filters out variation that is not directly related to the response and produces models of clearer interpretation, focusing the predictive information in one component, as shown in several recent studies of metabolomics [21,54]. The further improvements made by the OPLS-DA in MVA resides in the ability to separate the portion of the variance useful for predictive purposes from the not predictive variance (which is made orthogonal) [55]. OPLS-DA models are useful tools in application of prediction and classification. In order to evaluate the robustness and predictive ability of the statistical models, a seven-fold cross-validation procedure was performed [50,56,57]. The PCA and OPLS-DA models were validated using the internal cross-validation default method (seven-fold) and further evaluated with a permutation test (400 permutations), all available in the SIMCA-P software [58]. The quality of the models was described by R^2^ and Q^2^ parameters [58]. The first (R^2^) is a cross-validation parameter defined as the portion of data variance explained by the models and indicates the goodness of fit. The second (Q^2^) represents the portion of variance in the data predictable by the model. In our MVA 3–5 components usually gave optimal R^2^(cum) and Q^2^(cum) values. The minimal number of components required can be easily defined since R^2^(cum) and Q^2^(cum) parameters display a completely diverging behavior as the model complexity increases [58,59]. The results were shown by the optimal bi-dimensional scores plots and relative loadings plots, which were used to identify the molecular components responsible of separation among groups [21,60,61].

Furthermore, for both aqueous and lipid extracts, variation in discriminating metabolites content for each compartment, among the several samplings (March, May, and July) was determined by analyzing selected distinctive unbiased NMR signals after spectra normalization. The significant differences of the mean values were analyzed by analysis of variance (one-way ANOVA), with Tukey’s honestly significant difference (HSD) post hoc test, using the R statistical environment, Version 3.2.4, on a 64 bit Windows machine [62]. The levels of statistical significance were at *p*-values < 0.05 with a 95% confidence level.

## 5. Conclusions

In recent years, research has focused on the study of bioactive compounds from living organisms, in particular marine organisms. Among Cnidaria phylum, jellyfish represent a potentially new source of advantageous products for human health. *R. pulmo* represents one of the most abundant species of the Mediterranean coasts, with marked inter-annual fluctuations in population density. In this study, the metabolic profiles of the different body compartments (female gonads, umbrella, and oral arms) of *R. pulmo* from different sampling periods, were evaluated. The NMR analysis of the metabolic profiles of body fractions revealed that female gonads had a higher content in metabolites, while a similar biochemical composition was found in umbrella and oral arms. The analysis performed on both lipid and aqueous extracts showed that female gonads had the richest fraction, essentially due to the presence of ω-3 PUFAs (EPA and DHA), free amino acids (essential and not), and osmolytes (homarine and betaine). The results also indicated that the different relative content of these compounds might be due to variations in size and egg maturation of individuals and/or the temperature of the living biota and habitat, as already observed in other species. The presence of essential metabolites (not only for marine organisms but also for human health) in the gonads of *R. pulmo* suggests their potential use as a source for these compounds. Although further studies are required in this specific field, the obtained preliminary data on biochemical composition on *R. pulmo*, suggest several applications of these invertebrates in the fields of nutraceuticals, nutricosmetics, and functional food.

## Figures and Tables

**Table 1:** Chemical shift and assignments of main peaks in the ^1^H NMR spectra of CD_3_OD/CDCl_3_ extracts of the three different body compartments of *R. pulmo* (d: doublet; dd: double of doublets; t: triplet; m: multiplet; s: singlet).

Compound	Assignment	^1^H (ppm, Multiplicity)
Cholesterol (CHO)	-CH_3_-18	0.68–0.69 (s)
	-CH_3_-21	0.92 (d)
	-CH_3_-19	1.01 (s)
All fatty acids (STA, MUFA, DUFA) except ω-3 FA	-CH_3_	0.80–0.91 (t)
ω-3 PUFAs	-CH_3_	0.98 (t)
All fatty acids	-(CH_2_)_n_-	1.22–1.34 (m)
All fatty chains except DHA and EPA	COOCH_2_CH_2_-	1.57–1.66 (m)
EPA	COOCH_2_CH_2_-	1.67–1.74 (m)
All fatty acids except DHA	-CH=CH-CH_2_-	1.99–2.17 (m)
All fatty acids except DHA	COOCH_2_-	2.27–2.32 (t)
DHA	COOCH_2_CH_2_-	2.38 (dd)
DUFA/PUFA	-CH=CH-CH_2_--CH=CH-	2.77 (t)
PUFA (DHA, EPA)	-CH=CH-CH_2_--CH=CH-	2.80–2.85 (t)
PE	-CH_2_-N	3.03 (s)
PC and Sphingolipids	-(CH_3_)_3_-N	3.22 (overlapped s)
TG	-CH_2_- (sn-1,3)	4.11–4.14 (dd)
	-CH- (sn-2)	5.24 (m)
All MUFAs, DUFAs, PUFAs	-CH=CH-	5.28–5.43 (m)
1,2-DAGs	-CH-	5.26–5.13 (m)
	-CH_2_-	4.28–4.12 (m)
Homarine	-C3H-	8.61 (d)
	-C5H-	8.44 (dd)
	-C6H-	8.09 (d)
	C4H-	7.85 (dd)

**Table 2:** Chemical shifts and assignments of main peaks in the ^1^H NMR spectra of D_2_O extracts of the three different body compartments of *R. pulmo* (d: doublet; dd: double of doublets; t: triplet; m: multiplet; s: singlet). ^a^ Essential amino acid; TMAO: trimethylamine-N-oxide.

Compound	Assignment	^1^H (ppm, Multiplicity)
Leucine ^a^	-CH_3_	0.96 (d)
	-CH-	1.70 (m)
Isoleucine ^a^	-CH_3_	1.01 (d)
	-CH-	1.97 (m)
Valine ^a^	-CH_3_	1.05 (d)
	-CH-	2.29 (m)
Lactate	-CH_3_	1.33 (d)
	-CH-	4.16 (q)
Threonine ^a^	-CH_3_	1.33 (d)
	-CH-	3.68 (m)
	-CH-	4.29 (m)
Alanine	-CH_3_	1.48 (d)
	-CH-	3.79 (m)
Acetate	CH_3_	1.92 (s)
Proline	-CH_2_-	2.1–2 (m)
	-CH_2_-	2.32–2.36 (m)
	-CH_2_-	3.3–3.4 (m)
	-CH-	4.10–4.14 (m)
Glutamate	-CH_2_-	2.07 (m)
	-CH_2_-	2.36 (m)
Succinate	-CH_2_-	2.41 (s)
β-Alanine	-CH_2_-	2.56 (t)
	-CH_2_-	3.20 (t)
Hypotaurine	-CH_2_NH	3.27 (s)
	-CH_2_SO_3_	3.90 (s)
Unknown		2.73
		3.45
Unknown		2.92
		4.7
Betaine	-N(CH_3_)_3_	3.27 (s)
	-CH_2_-	3.90 (s)
TMAO	N(CH_3_)	3.27 (s)
Taurine	-CH_2_NH	3.27 (t)
	-CH_2_SO_3_	3.41 (t)
Glycine	-CH	3.57 (s)
Homarine	-N(CH_3_)_3_	4.37 (s)
	-C4H-	7.97 (dd)
	-C6H-	8.04 (d)
	-C5H-	8.55 (dd)
	-C3H-	8.71 (d)
Tyrosine	-C3,5H- ring	6.90 (d)
	-C2,6H- ring	7.19 (d)
Trigonelline	-C4H-	8.08 (dd)
	-C3H- and -C5H-	8.84 (dd)
	-C2H-	9.13 (s)
Formate	-CH	8.46 (s)

## Supplementary Materials

The following are available online, Figure S1: (a) t[1]/t[2] PCA scores plot for lipid extracts (R^2^X = 0.95, Q^2^ = 0.92) of three body compartments of *R. pulmo*. (b) t[1]/t[2] PCA scores plot for aqueous extracts (R^2^X = 0.73, Q^2^ = 0.53) of three body compartments of *R. pulmo*. Red circle, female gonads; blue square, oral arms; green triangle, umbrella.

## Abbreviations

FA	fatty acid
SFA	saturated fatty acid
MUFA	mono unsaturated fatty acid
DUFA	di-unsaturated fatty acid
PUFA	polyunsaturated fatty acid
TG	triglycerides
CHO	cholesterol
PC	phosphatidycholine
PE	phosphatidylethanolamine
EPA	eicosapentaenoic acid
DHA	docosahexaenoic acid
1,2 DAG	1,2 diacylglycerols
TMAO	trimethylamine-N-oxide
PCA	principal component analysis
OPLS-DA	orthogonal partial least squares discriminant analysis
