# Supplementary material for: 1H NMR Metabolic Profile of Scyphomedusa Rhizostoma pulmo (Scyphozoa, Cnidaria) in Female Gonads and Somatic Tissues: Preliminary Results
Source: Molecules. 2020 Feb 13;25(4):806. doi: 10.3390/molecules25040806 (PMC7070884; doi:10.3390/molecules25040806)
Supplement: Supplementary file 1 [file molecules-25-00806-s001.pdf]

Supplementary Information

^{1}H NMR Metabolic profile of scyphomedusa *Rhizostoma pulmo* (Scyphozoa, Cnidaria) in female gonads and somatic tissues: preliminary results

Angilè, F.[1], Del Coco L.[1], Girelli, C.R.[1], Basso, L.[1,2], Rizzo, L.[2,3], Piraino, S.[1,2], Stabili, L.[1,4], Fanizzi, F.P.[1*]

[1] Department of Biological and Environmental Sciences and Technologies (Di.S.Te.B.A.), University of Salento, via Monteroni, 73100- Lecce (Italy); federica.angile@unisalento.it; laura.delcoco@unisalento.it; chiara.girelli@unisalento.it; fp.fanizzi@unisalento.it

[2] CoNISMa, Piazzale Flaminio, 9, Roma (Italy); lorena.basso@unisalento.it; stefano.piraino@unisalento.it

[3] Integrative Marine Ecology, Stazione Zoologica Anton Dohrn, Villa Comunale, 80121 Napoli, (Italy); lucia.rizzo@szn.it

[4] Water Research Institute of the National Research Council, (IRSA-CNR), Via Roma 3, Taranto, (Italy); loredana.stabili@irsa.cnr.it

* Correspondence: fp.fanizzi@unisalento.it; tel.: +39-0832-299265

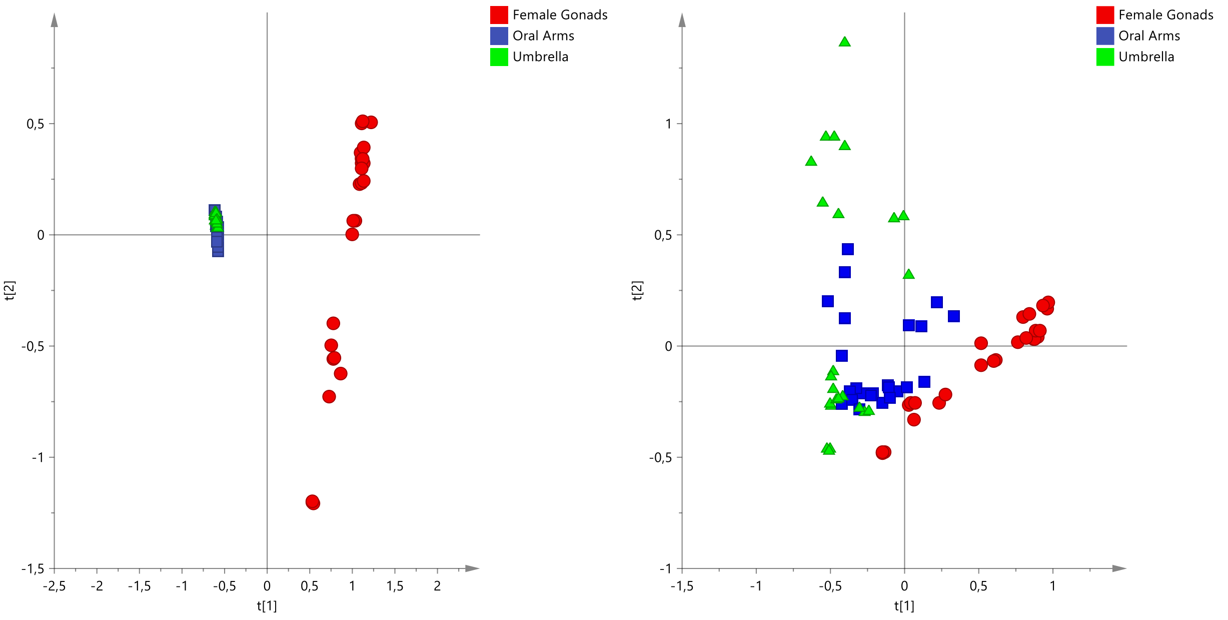

Figure S1. a) t[1]/t[2] PCA scores plot for lipid extracts (R^2X=0.95, Q^2=0.92) of three body compartments of *R. pulmo*. **b)** t[1]/t[2] PCA scores plot for aqueous extracts (R^2X=0.73, Q^2=0.53) of three body compartments of *R. pulmo*. Red circle, female gonads; blue square, oral arms; green triangle, umbrella.
